# Maternal nutrition alters mRNA isoform expression, usage, and splicing dynamics in skeletal muscle of beef cattle offspring

**DOI:** 10.3389/fgene.2026.1734234

**Published:** 2026-02-05

**Authors:** Guilherme Henrique Gebim Polizel, Ángela Cánovas, Germán D. Ramírez-Zamudio, Aline Silva Mello Cesar, Wellison J. S. Diniz, José Bento Sterman Ferraz, Heidge Fukumasu, Luiz F. Brito, Felipe Eguti de Carvalho, Miguel Santana

**Affiliations:** 1 Department of Animal Science, Faculty of Animal Science and Food Engineering, University of São Paulo, Pirassununga, Brazil; 2 Centre for Genetic Improvement of Livestock (CGIL), Department of Animal Biosciences, University of Guelph, Guelph, ON, Canada; 3 Department of Food Science and Technology, Luiz de Queiroz College of Agriculture, University of São Paulo, Piracicaba, Brazil; 4 Department of Animal Sciences, College of Agriculture, Auburn University, Auburn, AL, United States; 5 Department of Animal Sciences, Purdue University, West Lafayette, IN, United States

**Keywords:** amino acid transport, fetal programming, gene and transcript expression, regulatory mechanisms, SLC gene family, splicing events

## Abstract

**Background:**

Maternal nutrition during gestation plays a critical role in fetal muscle development and long-term metabolic programming; however, its persistent molecular effects on offspring skeletal muscle remain unclear. Therefore, the main objective of this study was to investigate the influence of prenatal nutrition on long-term differential gene expression (DGE), differential mRNA transcript expression (DTE), and differential transcript usage (DTU) in skeletal muscle of beef cattle.

**Methods:**

A total of 126 pregnant Nellore cows were assigned to three dietary treatments: mineral supplementation only (NP), protein-energy supplementation during late gestation (PP), and protein-energy supplementation throughout gestation (FP). At 676 ± 28 days of age, muscle samples were collected from offspring for RNA sequencing. The DGE and DTE analyses were performed using the edgeR package, while DTU was evaluated with the IsoformSwitchAnalyzeR package. Over-representation analysis was conducted using g:Profiler.

**Results:**

A total of 27,412 genes and 111,185 transcripts, including novel loci and isoforms were identified. Gene-level differences were modest (16 genes), whereas transcript-level analyses revealed stronger effects, with a higher number of significant expression and usage changes across conditions. The FP × NP comparison exhibited the greatest impact on gene expression, with 14 DTEs and 87 DTUs, compared with 12 and 30 in PP × NP, and 10 and three in FP × PP, respectively. Isoform switching was observed in key genes including *SLC7A8*, *SLC25A30*, *SORBS3*, and *CDH13* genes, influencing coding potential, functional domains, and mRNA stability, with potential consequences for amino acid transport, cytoskeletal organization, and muscle regeneration. Functional enrichment analyses highlighted significant metabolic pathways related to amino acid and biotin metabolism, intracellular trafficking, and immune regulation.

**Conclusion:**

Overall, prenatal nutrition, particularly protein-energy supplementation throughout gestation in comparison to mineral supplementation, modulates offspring muscle mainly through transcript usage and splicing, suggesting long-term adaptive mechanisms beyond gene-level regulation.

## 1 Introduction

Beef is a major source of high-quality protein, essential fatty acids, and trace minerals, playing a critical role in human health and nutrition (Murimi, 2022). However, improving beef quality remains a challenge for the cattle sector (da Silveira et al., 2021) and continues to be a central focus in beef cattle breeding and nutritional genomics research. Among the extrinsic factors influencing meat quality, maternal nutrition has been suggested to play an important role in muscle development and meat characteristics in the offspring, potentially exerting long-term effects (Du et al., 2015). This is particularly relevant given that skeletal muscle, the primary tissue contributing to meat yield, starts its development during the intrauterine period; thus, optimizing fetal muscle development may enhance both production efficiency and overall beef quality (Yan et al., 2013; Du et al., 2015; Du et al., 2017; Costa et al., 2021).

During fetal development, the total number of skeletal muscle fibers is determined exclusively within the first two-thirds of gestation and is highly sensitive to maternal nutrition and other uterine environmental factors (Du et al., 2010). As skeletal muscle has lower priority in nutrient partitioning compared to vital organs such as the brain and liver, it is particularly vulnerable to maternal nutritional fluctuations (Long et al., 2009; Meyer et al., 2010). Phenotypic variations related to skeletal muscle tissue, including body morphology, body weight, growth rate, *longissimus dorsi* area, intramuscular fat (marbling content), and tenderness, observed postnatally originate from developmental alterations that occur during the prenatal period (Summers et al., 2011; Copping et al., 2021; Polizel et al., 2021b; Dean et al., 2024; Pombo et al., 2025). These outcomes are associated with epigenetic modifications established during gestation, which may induce long-lasting changes in gene expression throughout postnatal life and, in some cases, be inherited across generations (Shokrollahi et al., 2025).

Several recent studies have investigated the impact of prenatal nutrition on the muscle transcriptome (Diniz et al., 2021; Palmer et al., 2021; Carvalho et al., 2022; Shokrollahi et al., 2024; Keogh et al., 2025). However, most of these studies have focused primarily on gene-level expression analyses, which while informative, may overlook subtle yet functionally important differences in transcript-level dynamics (i.e., the expression levels of individual mRNA isoforms and in the relative usage of alternative splice transcripts (Muniz et al., 2021; Asselstine et al., 2022; Singh and Ahi, 2022). Changes in transcript usage (i.e., the relative proportion of mRNA isoforms expressed from a single gene) and mRNA isoform switching (i.e., a change in which isoform is predominantly expressed) can result in substantial downstream effects on protein structure, cellular metabolism, and ultimately, phenotypic outcomes (Wang et al., 2008; Pal et al., 2011; Jiang et al., 2023).

Here, we hypothesized that prenatal nutrition modulates the muscle transcriptome of beef cattle offspring at the isoform level, primarily through alternative splicing (AS) mechanisms. Therefore, the overarching objective of this study was to investigate the long-term effects of maternal nutrition during gestation on muscle transcript expression and transcript usage in both novel and known genes and transcripts. The specific objectives were to: (1) identify novel beef muscle genes and transcripts; (2) evaluate differential gene- and transcript-level expression profiles among offspring from contrasting maternal nutrition groups; (3) characterize transcript usage patterns, mRNA isoform switches, and AS events associated with maternal nutrition; and, (4) perform functional enrichment analyses to elucidate the molecular pathways and biological processes underlying the observed transcriptomic differences.

## 2 Materials and methods

### 2.1 Experimental design

This study is part of a broader research initiative to evaluate the long-term molecular effects of maternal nutrition on the development and performance of beef cattle offspring. The experimental animals were managed at the Faculty of Animal Science and Food Engineering (FZEA-USP) campus (Pirassununga, SP, Brazil). The Research Ethics Committee of FZEA-USP approved the study under protocol number 1843241117, in accordance with the ethical standards and guidelines of the National Council for the Control of Animal Experimentation.

The nutritional interventions applied to 126 pregnant Nellore cows during gestation were previously described by Schalch Junior et al. (2022). Briefly, cows were artificially inseminated (AI) using semen from four sires, and pregnancy was confirmed 30 days post-AI. The dams were then stratified into three experimental groups (n = 42 per group), balanced for age, body weight (BW), and body condition score. Each group was maintained in grazing paddocks with *Urochloa brizantha* cv. Marandu, which were equipped with troughs for water and feed supplementation. The cows were assigned to one of three prenatal nutritional treatments: (1) NP (Not Programmed; control) – mineral supplementation only (0.3 g/kg BW per day) throughout gestation; (2) PP (Partially Programmed) – protein–energy supplementation (3 g/kg BW per day) provided only during the third trimester; and, (3) FP (Fully Programmed) – the same protein–energy supplementation (3 g/kg BW per day) provided continuously from pregnancy confirmation until calving. All groups received mineral supplementation at 0.3 g/kg of BW per day; however, in the PP and FP groups, the minerals were incorporated into the protein–energy supplement formulation (Table 1). The quality of the grazing areas was monitored through periodic bromatological analyses to ensure comparable nutrient availability among treatments. The total digestible nutrient (TDN) content averaged 63.07%, 64.1%, and 61.43% for the NP, PP, and FP groups, respectively, while crude protein (CP) levels were 7.38%, 7.82%, and 7.40%, respectively. Further methodological details and the effects of maternal nutritional treatments on dam performance and metabolism are provided in Schalch Junior et al. (2022).

**TABLE 1:** Composition of the different supplements offered to the Nellore cows during the gestational period.

Ingredients	Mineral supplement	Protein-energy supplement
Corn (%)	35.00	60.00
Soybean meal (%)	-	30.00
Dicalcium phosphate (%)	10.00	-
Urea 45% (%)	-	2.50
Salt (%)	30.00	5.00
Minerthal 160 MD (%)^a^	25.00	2.50
Total digestible nutrients (%)	26.76	67.55
Crude protein (%)	2.79	24.78
Non-protein nitrogen (%)	-	7.03
Acid detergent fiber (%)	1.25	4.76
Neutral detergent fiber (%)	4.29	11.24
Fat (%)	1.26	2.61
Calcium (g/kg)	74.11	6.20
Phosphorus (g/kg)	59.38	7.24

^a^ Mineral premix composition (Minerthal company; Goiânia, Goiás, Brazil): Calcium = 8.6 g/kg; Cobalt = 6.4 mg/kg; Copper = 108 mg/kg; Sulfur = 2.4 g/kg; Fluorine = 64 mg/kg; Phosphorus = 6.4 g/kg; Iodine = 5.4 mg/kg; Manganese = 108 mg/kg; Selenium = 3.2 mg/kg; Zinc = 324 mg/kg; Sodium monensin = 160 mg/kg (Polizel et al., 2021a).

**FIGURE 1:**
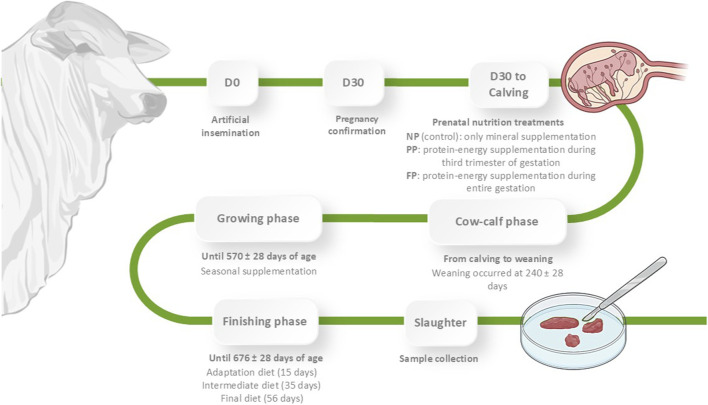
Schematic overview of the experimental design and nutritional treatments (NP, PP, and FP) applied during gestation and postnatal development in Nellore cattle.

After calving, protein–energy supplementation for the PP and FP dams was discontinued. Thereafter, all groups, including NP, received 0.3 g/kg BW of mineral supplement daily and were managed together in a single grazing system with *Urochloa brizantha* cv. Marandu. Standardized health and feeding protocols were followed for cow–calf management until weaning at 240 ± 28 days. Post-weaning, calves were separated by sex and reared under uniform management conditions until reaching 570 ± 28 days of age. The young bulls received two types of supplements depending on the season: an energy supplement during the dry season and a protein supplement during the wet season, while continuing to graze on the same pasture. The finishing phase began at 570 ± 28 days of age and included 63 bulls, lasting until slaughter at 676 ± 28 days of age. During this period, the animals received three sequential diets: a 15-day adaptation diet, a subsequent diet for 35 days, and a final diet for 56 days (Polizel et al., 2022a). At the end of the experimental period, all animals were slaughtered at the FZEA-USP slaughterhouse, following the regulations established by the Brazilian Ministry of Agriculture, Livestock, and Food Supply (MAPA; Brasilia, DF, Brazil). Figure 1 illustrates a schematic overview of the experimental design.

### 2.2 Collection of muscle samples

Immediately after slaughter, samples of the *longissimus thoracis* muscle (located between the 12th and 13th ribs) were collected from all bulls for transcriptomic analyses. Tissue samples were obtained within 15 min postmortem, rapidly snap-frozen in liquid nitrogen, and stored at −80 °C until RNA extraction. From the 63 bulls, a subset of 15 animals (n = 5 per maternal nutrition group), all sired by the same bull (to minimize paternal genetic variation), was randomly selected for RNA sequencing (RNA-Seq) analyses.

### 2.3 RNA isolation, library preparation, and sequencing

Total RNA was extracted from 100 mg of *longissimus thoracis* muscle using TRIzol reagent (Thermo Fisher Scientific, Waltham, MA, United States), following the manufacturer’s instructions. RNA concentration and purity were assessed with a DS-11 spectrophotometer (DeNovix, Wilmington, DE, United States), and RNA integrity was evalauted with the Bioanalyzer 2,100 (Agilent Technologies, Santa Clara, CA, United States). Only samples with a RNA Integrity Number (RIN) > 7.0 were retained for subsequent analyses.

For library construction, 0.1–1 µg of high-quality RNA was used following the TruSeq Stranded mRNA Sample Preparation protocol (Illumina, San Diego, CA, United States). Library quantification was performed via quantitative PCR (qPCR) using the KAPA Library Quantification kit (Roche, Basel, Switzerland), and average fragment size was determined with the Bioanalyzer. Sequencing was performed on a single flow-cell lane using the TruSeq PE Cluster kit v3-cBot-HS and a paired-end sequencing configuration on the Illumina HiSeq 2,500 platform. All sequencing procedures were conducted by NGS Soluções Genômicas (Piracicaba, São Paulo, Brazil).

### 2.4 Mapping, assembly, classification, and quantification of novel and known transcripts

Quality control of the raw RNA-Seq reads was performed using FastQC (v0.12.1), and adapter sequences as well as low-complexity reads were removed with SeqyClean (v1.10.09; Zhbannikov et al., 2017). The cleaned reads were then aligned to the *Bos taurus* reference genome (ARS-UCD1.3) using the STAR aligner (version 2.7.11b; Dobin et al., 2013) in two-pass mode (Veeneman et al., 2016) based on the Ensembl genome annotation (release 113) database. The resulting coordinate-sorted BAM files (“Aligned.sortedByCoord.out.bam”) were assembled into transcripts using StringTie (v.2.1.7; Pertea et al., 2015) with default parameters. Subsequently, the transcriptomes from all the samples were merged using the StringTie “*merge*” function to produce a unified transcriptome assembly. By default, StringTie assigns the prefix “MSTRG” to novel genes and transcripts.

To classify known and novel transcripts, the unified GTF file was compared to the *Bos taurus* reference annotation (ARS-UCD1.3.113) using GffCompare (v.0.12.10; Pertea and Pertea, 2020). Predicted transcripts were categorized according to GffCompare class codes as follows: known isoforms of a reference gene (class code “=”), novel isoforms of a reference gene (class codes “c”, “k”, “j”, “m”, “n”, or “o”), novel loci (class codes “i”, “u”, “y”, or “x”), and, potential artifacts (class codes “e”, “s”, or “p”) (Halstead et al., 2021).

The GffRead tool (v.0.12.7; Pertea and Pertea, 2020) was used to generate a transcriptome FASTA file from the StringTie-assembled GTF file and the *Bos taurus* reference genome FASTA. This custom transcriptome assembly was then indexed and used for transcript quantification with Salmon (v1.10.1; Patro et al., 2017) operating in quasi-mapping mode to estimate transcript-level abundance.

### 2.5 Differential gene expression (DGE) and differential transcript expression (DTE) analyses

Transcript-level counts from Salmon were imported into R (v4.4.1) using the tximport package (v.1.32.0; Soneson et al., 2016). A DGEList object was then created using edgeR (v.4.2.2; Robinson et al., 2010). Lowly expressed transcripts were filtered out using the “filterByExpr” function and 39,280 transcripts were kept for subsequent analyses. Data normalization was performed using the Trimmed Mean of M-values (TMM) method based on library sizes. Differential expression analyses were conducted in edgeR using the exact test based on the negative binomial distribution. The analysis included the prenatal nutritional group as the only factor of interest. Statistical significance was assessed using the “exactTest” function, and p-values were adjusted for multiple testing using the false discovery rate (FDR) approach.

The normalized counts were used for differential expression (DE) analysis, whereas log_2_-transformed counts per million (CPM) values were used for performing principal component analysis (PCA) using the “prcomp” R function. For gene-level analyses, the same computational workflow was employed, but gene expression matrices were obtained using the “extractGeneExpression” function from the IsoformSwitchAnalyzeR package (v.2.2.0; Vitting-Seerup et al., 2019). After filtering, 15,832 genes were retained for downstream analyses. Transcripts and genes were considered significantly differentially expressed when the FDR-adjusted p-value was lower than 0.05 and the absolute log_2_ fold change (|log_2_FC|) exceeded 1.

### 2.6 Differential transcript usage (DTU)

The pipeline of IsoformSwitchAnalyzeR (Vitting-Seerup et al., 2019) was used to investigate differential transcript usage (DTU). Only genes with two or more isoforms (“removeSingleIsoformGenes” = TRUE), an average expression >10 transcripts per million (TPM; “geneExpressionCutoff” = 10) across experimental groups, and at least one isoform expressed >3 TPM (“isoformExpressionCutoff” = 3) in at least one group were retained. After filtering, 7,973 isoforms remained for downstream analyses.

Isoform switching in each contrast (FP × NP, FP × PP, and NP × PP) was assessed using the DEXSeq method (Anders et al., 2012), which implements a generalized linear model (GLM) framework based on the negative binomial distribution to test for differences in relative transcript (or exon) usage between conditions. In this approach, count data from transcript regions are modeled so that changes in relative usage across groups can be detected while accounting for biological variability. DEXSeq fits models with and without condition–region (exon) interactions and performs likelihood ratio tests to identify significant differential usage, with p-values adjusted for multiple testing using the false discovery rate (FDR) method. The exon- or region-level differential usage results are subsequently integrated within the IsoformSwitchAnalyzeR framework to infer transcript-level (isoform) usage patterns. The difference in isoform fraction (dIF) was calculated as IF_2_ – IF_1_, where IF_1_ and IF_2_ represent the isoform fractions of the first and second groups in each contrast, respectively (e.g., FP in FP × NP or FP × PP). The dIF value serves as an estimate of effect size, analogous to fold change in differential expression analysis. Isoform switches were considered as significant when |dIF| > 0.1 and FDR <0.05.

Further analyses of the switched isoforms were performed to evaluate their potential consequences on gene function, including intron retention (IR), changes in the amino acid sequences of open reading frames (ORFs), susceptibility to nonsense-mediated decay (NMD), modifications in protein domain structures, and variations in coding potential. A comprehensive examination of notable isoform switches was conducted using multiple bioinformatics tools: IUPred2A web server (Mészáros et al., 2018) for predicting intrinsically disordered regions; SignalP (v.5.0; Teufel et al., 2022); for signal peptide prediction; CPC2 (v.1.0.1; Kang et al., 2017) for coding potential evaluation; Pfam (v.37.0; Finn et al., 2014); for identifying conserved protein domains; DeepTMHMM (v.1.0.44; Hallgren et al., 2022); for protein topology prediction; and DeepLoc2 (v.2.0; Thumuluri et al., 2022); for subcellular location inference. In addition, AS events associated with isoform switching were investigated, including alternative 3′ and 5′ splice sites (A3, A5), alternative transcription start and termination sites (ATSS, ATTS), exon skipping (ES), and intron retention (IR). These events can reveal potential modifications in gene function mediated by altered exon junctions and transcript structural variation.

### 2.7 Over-representation analysis (ORA) and overlapping transcripts and processes

**FIGURE 2:**
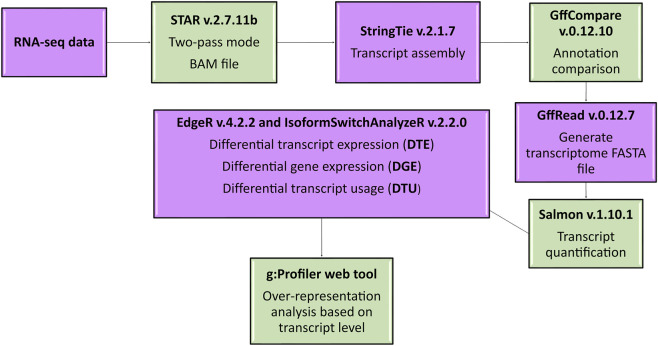
Bioinformatics workflow to identify and characterize genes, transcripts and processes relevant to prenatal nutrition in offspring muscle.

As the primary objective of this study was to evaluate the effects of maternal nutrition at the transcript level, rather than through conventional gene-level analyses, the over-representation analysis (ORA) was performed using transcript-based data. Genes corresponding to significant transcripts identified in differential transcript expression (DTE) or DTU analyses were used as input for enrichment testing. ORA was conducted separately for each contrast (FP × PP, PP × NP, and FP × NP) and for both analysis type (DTE and DTU). Functional enrichment was carried out using Gene Ontology (GO) analysis including the three main GO categories: Biological Process (BP), Molecular Function (MF), and Cellular Component (CC). Furthermore, metabolic pathways analyses were performed using the Reactome (REAC) and Kyoto Encyclopedia of Genes and Genomes (KEGG) databases via the g:Profiler platform (Kolberg et al., 2023). Enriched processes were considered significant when FDR was lower than 0.05. The overlap of significant transcripts between DTE and DTU analyses, as well as the intersection of enriched biological processes, was visualized using the R packages “eulerr” (v7.0.2) and “ggvenn” (v0.1.10). A schematic overview of the main bioinformatics workflow is presented in Figure 2.

## 3 Results

### 3.1 Classification of muscle genes and transcripts

**FIGURE 3:**
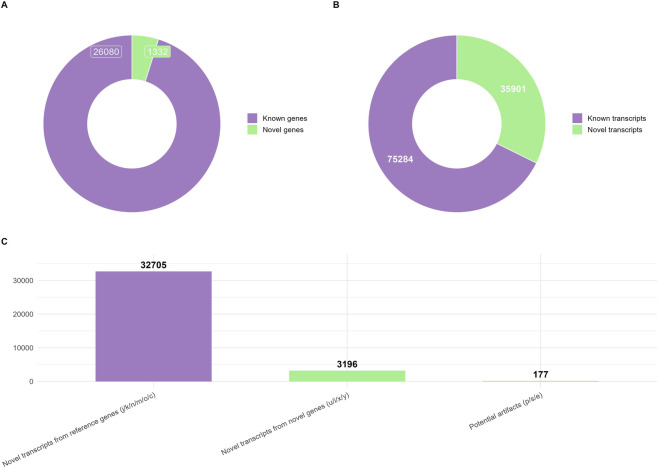
Classification of the transcripts and genes identified. **(A)** Proportion of known and novel genes. **(B)** Proportion of known and novel transcripts. **(C)** Categories of novel transcripts identified.

A total of 27,412 genes were identified in the assembled muscle transcriptome, including 26,080 known genes and 1,332 novel genes (Figure 3A). At the transcript level, 111,185 transcripts were detected, of which 75,284 transcripts were classified as known and 35,901 as novel (Figure 3B). Among the novel transcripts, 32,705 originated from reference genes, 3,196 from novel genes, and 177 were categorized as potential artifacts (Figure 3C). Comprehensive details for each gene and transcript are provided in the Supplementary Material: Additional File 1 (known transcripts), Additional File 2 (novel loci), Additional File 3 (novel transcripts from reference genes), and Additional File 4 (potential artifacts).

### 3.2 Principal component analysis (PCA) and differential expression of genes and transcripts (DGE and DTE)

**FIGURE 4:**
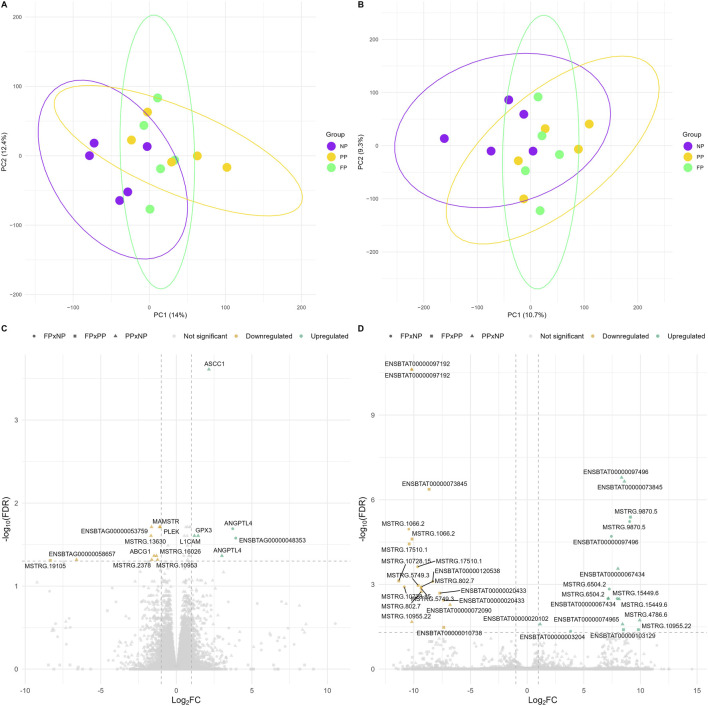
Principal component analysis (PCA) plot and volcano plot illustrating muscle gene and transcript profiles according to the maternal nutritional groups (NP, PP, and FP). **(A)** PCA of gene expression levels from different prenatal groups (NP, PP, and FP). **(B)** PCA of transcript expression levels from different prenatal groups (NP, PP, and FP). **(C)** Volcano plot of the differentially expressed genes across the group comparisons (FP × NP, FP × PP, and PP × NP). **(D)** Volcano plot of the differentially expressed transcripts across the group comparisons (FP × NP, FP × PP, and PP × NP).

The PCA of both gene-level (Figure 4A; PC1 = 14% and PC2 = 12.4%) and transcript-level (Figure 4B; PC1 = 10.7% and PC2 = 9.3%) expression data showed similar distribution patterns, with considerable overlap among the experimental groups. This overlap indicates the lack of a clear global distinction between the maternal nutrition treatments at either gene or transcript level.

Differential expression analysis enabled the identification of 13 DEGs between the PP and NP groups (FDR <0.05 and |log_2_FC| > 1), two genes between FP × NP, and one gene between FP × PP (Figure 4C; Additional File 5). At the transcript level (Figure 4D; Additional File 6), 14 transcripts were DE in the FP × NP contrast, 12 in PP × NP, and 10 in FP × PP. Notably, among the 13 DEGs, five corresponded to novel genes (*MSTRG.19105*, *MSTRG.2378*, *MSTRG.10953*, *MSTRG.16026*, and *MSTRG.13630*), while at the transcript level, 10 unique novel transcripts were identified (Figure 4).

### 3.3 Differentially transcript usage (DTU), functional consequences and splicing events

**FIGURE 5:**
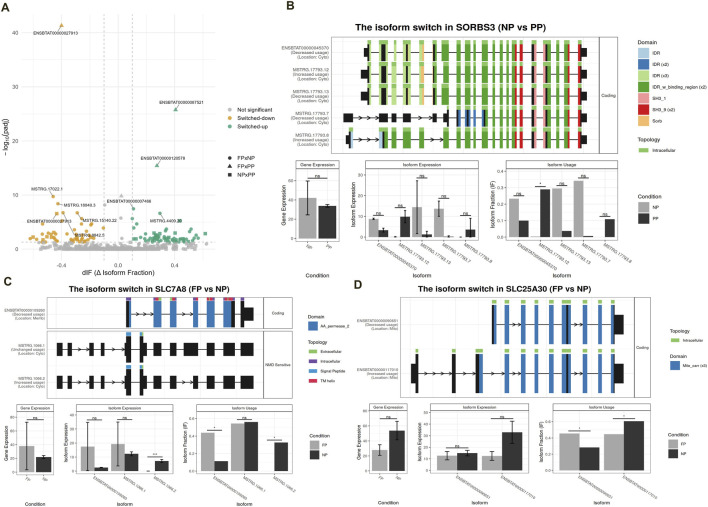
Differential transcript usage (DTU) analysis and main isoforms illustrated. **(A)** Volcano plot of DTU analysis across group comparisons (FP × NP, FP × PP, and NP × PP). **(B)** *SORBS3* gene in the NP × PP contrast showing the isoforms identified and results from DTE, DGE, and DTU analyses. **(C)** *SLC7A8* gene in the FP × NP contrast showing the isoforms identified and results from DTE, DGE, and DTU analyses. **(D)** *SLC25A30* gene in the FP × NP contrast showing the isoforms identified and results from DTE, DGE, and DTU analyses.

**FIGURE 6:**
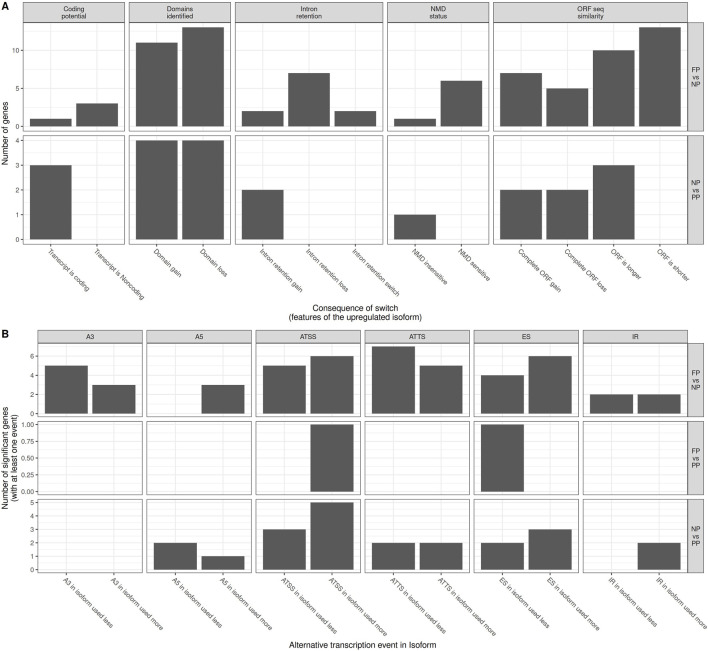
Number of genes in each contrast (FP × NP, FP × PP, and NP × PP) with **(A)** associated switch isoform consequences and **(B)** alternative splicing events.

A total of 120 transcripts exhibited DTU across the three maternal nutrition contrasts: NP × PP, FP × NP, and FP × PP (Figure 5A; Additional File 7). Among these, 30 transcripts were detected in NP × PP comparison, 87 in FP × NP, and three in FP × PP. We further assessed the functional impact of isoform switching for each significant isoform detected in the DTU analysis (Figure 6A; Additional File 8). Although some isoform switching events occurred in the FP × PP comparison, none resulted in detectable functional consequences (Figure 6A). In contrast, 21 switch consequences were detected in the NP × PP comparison, involving different domains, coding potential, ORF similarity, intron retention, and other features across 11 genes. For the FP × NP contrast, 93 switch consequences were detected, affecting 38 unique genes. Representative examples of switch consequences in key genes are illustrated in Figure 5B (*SORBS3* gene), Figure 5C (*SLC7A8* gene), and Figure 5D (*SLC25A30* gene). These genes were selected as illustrative cases because they showed robust and biologically meaningful isoform switching events (high dIF values and significant FDR), and because they are functionally linked to metabolic, transport, and cellular adaptation processes previously reported to be sensitive to maternal nutritional status. As such, they provide representative examples of how prenatal nutrition may influence transcript usage with potential functional consequences in offspring muscle. Figure 6A provides an overview of the number of genes exhibiting isoform switching with associated functional consequences across all pairwise comparisons.

AS events associated with isoform switching are shown in Figure 6B, with detailed information for each affected gene and isoform provided in Additional File 9. In the FP × NP contrast, the most frequent AS event was ATTS in the less-used isoform. For the FP × PP comparison, only two events AS were detected: ATSS in the more-used isoform and ES in the less-used isoform. Finally, in the NP × PP contrast, the predominant AS event was ATSS in the more-used isoform.

### 3.4 Over-representation analyses (ORA)

In the FP × NP comparison, ORA of the genes identified through the DTE analysis revealed significant enrichment of pathways related to biotin metabolism (GO:MF:0004077 [FDR = 0.0196], GO:MF:0009374 [FDR = 0.0196], and KEGG:00780 [FDR = 0.0226]). Additional enriched pathways were associated with transport of small molecules (REAC:R-BTA-382551 [FDR = 0.0260]), and with cell adhesion and cadherin regulation (REAC:R-BTA-9759476 [FDR = 0.0260], REAC:R-BTA-9764260 [FDR = 0.0260], REAC:R-BTA-9762292 [FDR = 0.0260], and REAC:R-BTA-9759475 [FDR = 0.0260]. The endosomal/vacuolar metabolic pathway was also significantly enriched (REAC:R-BTA-1236977, FDR = 0.0260).

The DTU analyses further revealed significant enrichment of pathways related to intracellular trafficking and vesicle-mediated transport (REAC:R-BTA-1236977 [FDR = 0.00047] and REAC:R-BTA-1236974 [FDR = 0.0021]), as well as cellular structural organization (GO:CC:0043229 [FDR = 0.0024], GO:CC:0031982 [FDR = 0.0024], GO:CC:0043226 [FDR = 0.0024], GO:CC:0110165 [FDR = 0.0035], GO:CC:0005622 [FDR = 0.0038], and GO:CC:0005575 [FDR = 0.0038]). In addition, immune-related pathways were significantly enriched (REAC:R-BTA-983169 [FDR = 0.0033] and REAC:R-BTA-983170 [FDR = 0.0033]).

In the FP × PP contrast, over-represented processes identified from the DTE analysis were predominantly associated with amino acid metabolism and transmembrane transport (GO:BP:0035524 [FDR = 0.0191], GO:BP:1903801 [FDR = 0.0191], GO:BP:1904273 [FDR = 0.0191], GO:BP:1904557 [FDR = 0.0191], GO:CC:0015827 [FDR = 0.0191], and GO:CC:0015829 [FDR = 0.0191]). Additional enriched processes were associated with inositol metabolism and biosynthesis (GO:MF:0004512 [FDR = 0.0109], GO:BP:0006021 [FDR = 0.0191], GO:BP:0006020 [FDR = 0.0191], and GO:MF:0016872 [FDR = 0.0109]).

**FIGURE 7:**
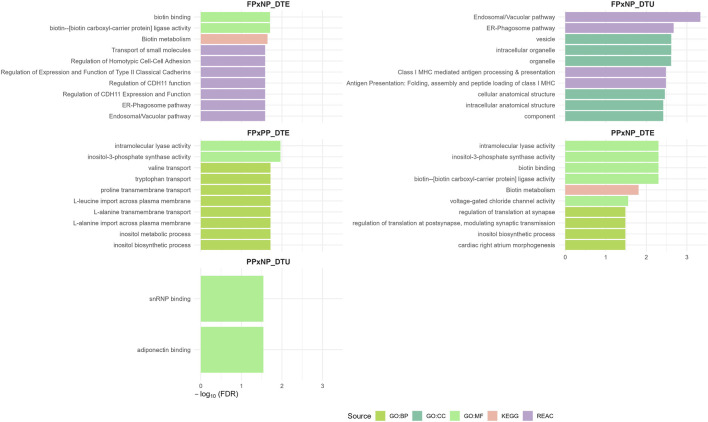
Over-representation analysis for each contrast (FP × NP, FP × PP, and PP × NP) and analysis type (DTE and DTU), highlighting significantly enriched metabolic pathways and GO terms associated with the significant transcripts. The y-axis shows enriched terms grouped by source (GO:BP, GO:MF, GO:CC, KEGG, and REAC), and the x-axis indicates the enrichment significance, expressed as–log10(FDR).

In the PP × NP contrast, genes identified through DTE analysis were predominantly enriched in pathways related to biotin metabolism (GO:MF:0004077 [FDR = 0.0050], GO:BP:0009374 [FDR = 0.0050], and KEGG:00780 [FDR = 0.0155]). Additional enriched biological processes were associated with inositol metabolism and biosynthesis (GO:MF:0004512 [FDR = 0.0050], GO:MF:0016872 [FDR = 0.0050], and GO:BP:0006021 [FDR = 0.0331]). Regarding DTU-based ORA, significant enrichment was observed for molecular functions related to adiponectin binding and small nuclear ribonucleoprotein (snRNP) binding (GO:MF:0055100 [FDR = 0.028] and GO:MF:0070990 [FDR = 0.028], respectively). A summary of all over-represented biological processes and molecular functions across the three nutritional contrasts and both analytical approaches (DTE and DTU) is presented in Figure 7. Complete details of the ORA are provided in Additional File 10.

### 3.5 Overlapping transcripts and processes across comparisons and analyses

**FIGURE 8:**
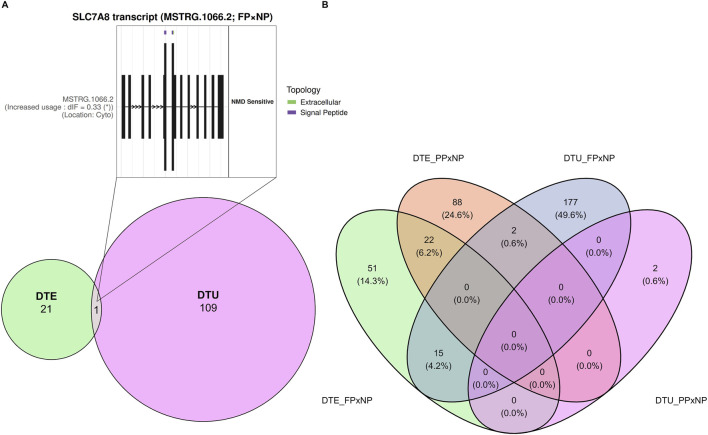
Exclusive and overlapping transcripts between analyses (DTE and DTU; **(A)**), and exclusive and overlapping enriched metabolic pathways and GO terms from comparisons with significant processes in both analyses (FP × NP and PP × NP; **(B)**).

Among the significant isoforms identified in both the DTE and DTU analyses, only *MSTRG.1066.2*, an isoform of the *SLC7A8* gene, was found to overlap between them (Figure 8A). The ORA also revealed processes shared across comparisons and analysis types (Figure 8B), with detailed information provided in Table 2. In the FP × NP comparison, the DTU and DTE analyses shared 15 over-represented processes, indicating convergence in transcript-level and isoform-usage responses to maternal nutrition. In contrast, no overlap was observed between DTU and DTE in the PP × NP comparison. As described in Section 3.4, no over-represented processes were identified for the DTU analysis in the FP × PP contrast; therefore, this comparison was excluded from the overlap assessment.

**TABLE 2:** Overlapping enriched terms across prenatal nutrition group comparisons with significant processes in both analyses (DTE and DTU).

Overlapping over-represented processes	Source	Contrasts
Biotin binding	GO:Molecular function	DTE_FP × NP/DTE_PP × NP
Biotin metabolism	KEGG	DTE_FP × NP/DTE_PP × NP
Biotin--[biotin carboxyl-carrier protein] ligase activity	GO:Molecular function	DTE_FP × NP/DTE_PP × NP
KEGG root term	KEGG	DTE_FP × NP/DTE_PP × NP
Modification of postsynaptic actin cytoskeleton	GO:Biological process	DTE_FP × NP/DTE_PP × NP
Modification of postsynaptic structure	GO:Biological process	DTE_FP × NP/DTE_PP × NP
Positive regulation of actin nucleation	GO:Biological process	DTE_FP × NP/DTE_PP × NP
Positive regulation of Arp2/3 complex-mediated actin nucleation	GO:Biological process	DTE_FP × NP/DTE_PP × NP
Positive regulation of neurotrophin TRK receptor signaling pathway	GO:Biological process	DTE_FP × NP/DTE_PP × NP
Regulation of Arp2/3 complex-mediated actin nucleation	GO:Biological process	DTE_FP × NP/DTE_PP × NP
Regulation of modification of postsynaptic actin cytoskeleton	GO:Biological process	DTE_FP × NP/DTE_PP × NP
Regulation of modification of postsynaptic structure	GO:Biological process	DTE_FP × NP/DTE_PP × NP
Regulation of modification of synaptic structure	GO:Biological process	DTE_FP × NP/DTE_PP × NP
Regulation of neurotrophin TRK receptor signaling pathway	GO:Biological process	DTE_FP × NP/DTE_PP × NP
Regulation of translation at postsynapse	GO:Biological process	DTE_FP × NP/DTE_PP × NP
Regulation of translation at postsynapse, modulating synaptic transmission	GO:Biological process	DTE_FP × NP/DTE_PP × NP
Regulation of translation at synapse	GO:Biological process	DTE_FP × NP/DTE_PP × NP
Regulation of translation at synapse, modulating synaptic transmission	GO:Biological process	DTE_FP × NP/DTE_PP × NP
Response to biotin	GO:Biological process	DTE_FP × NP/DTE_PP × NP
Translation repressor activity	GO:Molecular function	DTE_FP × NP/DTE_PP × NP
Voltage-gated chloride channel activity	GO:Molecular function	DTE_FP × NP/DTE_PP × NP
Voltage-gated monoatomic anion channel activity	GO:Molecular function	DTE_FP × NP/DTE_PP × NP
Adaptive immune system	REACTOME	DTE_FP × NP/DTU_FP × NP
Alanine transmembrane transporter activity	GO:Molecular function	DTE_FP × NP/DTU_FP × NP
Antigen presentation: Folding, assembly and peptide loading of class I MHC	REACTOME	DTE_FP × NP/DTU_FP × NP
Antigen processing-Cross presentation	REACTOME	DTE_FP × NP/DTU_FP × NP
Branched-chain amino acid transmembrane transporter activity	GO:Molecular function	DTE_FP × NP/DTU_FP × NP
Class I MHC mediated antigen processing and presentation	REACTOME	DTE_FP × NP/DTU_FP × NP
Endosomal/Vacuolar pathway	REACTOME	DTE_FP × NP/DTU_FP × NP
ER-phagosome pathway	REACTOME	DTE_FP × NP/DTU_FP × NP
Glycine transmembrane transporter activity	GO:Molecular function	DTE_FP × NP/DTU_FP × NP
Immunoregulatory interactions between a lymphoid and a non-lymphoid cell	REACTOME	DTE_FP × NP/DTU_FP × NP
L-alanine transmembrane transporter activity	GO:Molecular function	DTE_FP × NP/DTU_FP × NP
L-leucine transmembrane transporter activity	GO:Molecular function	DTE_FP × NP/DTU_FP × NP
Protein binding	GO:Molecular function	DTE_FP × NP/DTU_FP × NP
Thyroid hormone transmembrane transporter activity	GO:Molecular function	DTE_FP × NP/DTU_FP × NP
Toxin transmembrane transporter activity	GO:Molecular function	DTE_FP × NP/DTU_FP × NP
Protein metabolic process	GO:Biological process	DTE_PP × NP/DTU_FP × NP
Regulation of protein metabolic process	GO:Biological process	DTE_PP × NP/DTU_FP × NP

## 4 Discussion

Previous studies investigated how different maternal nutrition strategies affect differential gene expression across tissues and their functional implications for Nellore cattle offspring (Polizel et al., 2022b; Polizel et al., 2025b; Cracco et al., 2024). However, identifying AS events with biologically relevant regulatory effects remains poorly explored in cattle. To our knowledge, this is the first study in Nellore cattle to investigate the impact of maternal nutrition on differential transcript regulation and its functional consequences. Splicing can generate distinct isoforms of the same gene, which may differ in regulatory elements, coding potential, and functional domains. These alterations can modify protein structure, stability, subcellular localization, and interaction networks (Hallegger et al., 2010). This study was designed to detect long-term transcriptomic changes in AS patterns leading to DTU and DTE in response to maternal nutrition strategies during gestation, with a focus on the skeletal muscle of offspring at the end of the finishing phase. Although PCA of both gene- and transcript-level expression did not reveal a clear global separation among maternal nutrition treatments, several genes and transcripts were differentially expressed. This pattern is consistent with a fine-tuning of transcriptional regulation rather than broad transcriptomic reprogramming, which would be more likely under drastic nutritional conditions such as severe overnutrition or undernutrition.

Overall, the FP treatment exhibited the strongest transcriptomic response relative to the control (NP), as reflected by the higher number of differentially expressed (n = 14) and differentially used transcripts (n = 87). Among these, a single novel transcript (*MSTRG.1066.2*), an isoform of the *SLC7A8* gene overlapped between the two analyses. This transcript was completely absent in the FP group (IF = 0) while representing 32.6% of the total *SLC7A8* expression in the NP group. The DTE analysis confirmed this pattern, showing consistent downregulation in the same isoform in the FP group. Importantly, *MSTRG.1066.2* is annotated as nonsense-mediated decay (NMD)-sensitive, suggesting that it is normally targeted for degradation through the NMD pathway (Peltz et al., 1993). This finding implies that prenatal nutrition strategies can influence both isoform production and overall mRNA stability, as changes in AS may regulate the balance between functional and degradable transcripts (Kishor et al., 2019). Consequently, the suppression of this NMD-sensitive isoform in the FP group could contribute to stabilized gene expression by reducing the abundance of transcripts prone to degradation. In line with this, the main coding transcript of the *SLC7A8* gene (*ENSBTAT00000109260*) showed increased usage in the FP group relative to NP, supporting the hypothesis that the suppression of the nonfunctional isoform favored the upregulation of the coding isoform.

These findings highlight the importance of AS and transcript-specific regulation as additional layers of post-transcriptional control that extend beyond conventional gene-level expression, potentially impacting protein synthesis, cellular function, and metabolic efficiency. Among the genes showing notable isoform switching with predicted functional consequences, *SLC7A8* and *SLC25A30* were selected for further discussion due to their roles in nutrient transport and energy metabolism. The *SLC7A8* gene belongs to the solute carrier (SLC) superfamily, which includes more than 458 transport proteins responsible for mediating the transmembrane movement of diverse substrates, including amino acids, ions, and metabolites (Pizzagalli et al., 2021). Specifically, *SLC7A8* encodes a neutral amino acid transporter that facilitates the uptake of leucine (Leu), valine (Val), glycine (Gly), alanine (Ala), serine (Ser), glutamate (Glu), and cysteine (Cys) (Meixner et al., 2020). Experimental evidence in mice demonstrates that the deletion of *SLC7A8* confers protection against diet-induced obesity (DIO) and improves glucose metabolism, thereby reducing lipid accumulation (Pitere et al., 2022). In parallel, SLC25A30, a mitochondrial solute carrier, has been reported to be associated with fatty acid metabolism, adipogenesis, and lipid metabolic pathways (Gunawan et al., 2021). In the context of beef cattle, the increased usage of the coding isoforms of *SLC7A8* (ENSBTAT00000109260) and *SLC25A30* (ENSBTAT00000090651) observed in the FP group reflects transcript-level modulation of genes with established roles in amino acid transport and mitochondrial metabolism. *SLC7A8* encodes a neutral amino acid transporter involved in the uptake of branched-chain and other neutral amino acids that contribute to metabolic homeostasis and nutrient availability (Fotiadis et al., 2013; Pizzagalli et al., 2021), whereas *SLC25A30* belongs to the mitochondrial solute carrier family, whose members are central to lipid and energy metabolism (Palmieri, 2013). Although the present study does not include protein-level or functional validation, the observed transcript usage patterns are consistent with differences in carcass subcutaneous fat previously reported for the same experimental population (Fernandes et al., 2023), supporting the biological relevance of these transcriptomic changes while warranting further functional investigation.

Several overlapping biological processes found in the FP × NP comparison were associated with amino acid transport and other molecular transport activities, functions closely related to SLC family genes (Hediger et al., 2013). Similar observations were reported by Palmer et al. (2021), who demonstrated that prenatal nutrition influences amino acid transport pathways in the beef muscle transcriptome. Such pathways likely contribute to muscle development by regulating amino acid availability for protein synthesis, modulating energy metabolism, and ultimately affecting growth efficiency and meat quality in beef cattle.

In the PP × NP comparison, no overlapping transcripts or processes were identified between the DTE and DTU analyses; however, several functional implications were noteworthy. For instance, the novel transcript *MSTRG.17793.12*, originating from the *SORBS3* gene was affected by maternal supplementation during the final third of gestation and exhibited higher usage in the PP group compared with NP. Although this transcript is protein-coding, it differs from the downregulated isoform (*MSTRG.17793.7*) in two key aspects: it encodes a longer ORF and shows a domain gain. Specifically, the upregulated isoform contains the Sorbin (SoHo) domain, whereas the downregulated variant lacks it, suggesting a domain-dependent functional divergence. The *SORBS3* gene codes for the adaptor protein vinexin and has been demonstrated to be involved in signal transduction induced by growth factors as well as in the structure of the cytoskeleton (Kioka et al., 2002). Alterations in SoHo and SH3 domain containing 3 (*SORBS3*) were previously linked to epigenetic regulation in skeletal muscle (Day et al., 2017). Obesity increased promoter methylation and suppressed *SORBS3* expression, whereas weight loss following Roux-en-Y gastric bypass reversed these changes and improved metabolic outcomes, including glucose homeostasis (Day et al., 2017). Thus, the differential isoform usage observed may reflect domain-dependent regulatory shifts, in which the absence of the SoHo domain in the downregulated isoform could diminish cytoskeletal signaling and cellular responsiveness, whereas the upregulated isoform may restore or even modulate these functions through distinct molecular interactions.

Another important gene with functional relevance in the PP × NP comparison was *CDH13*. Among the four isoforms associated with this gene, the transcript *ENSBTAT00000079761* exhibited decreased usage in the PP group. Compared with the upregulated isoform (*ENSBTAT00000106688*), this transcript showed a loss of protein domains, suggesting a potential shift in functional capacity. The *CDH13* gene encodes the T-cadherin protein, a member of the cadherin superfamily involved in cell–cell adhesion, actin cytoskeleton organization, tissue morphogenesis, homeostasis, and angiogenesis (Hulpiau and van Roy, 2009; Rubina et al., 2021). Many of these functions are mediated through ligand binding, as T-cadherin serves as a receptor for adiponectin and LDL (Rubina et al., 2021). In skeletal muscle, adiponectin–T-cadherin signaling enhances muscle regeneration through the specific binding of adiponectin to T-cadherin (Tanaka et al., 2019). Therefore, the observed differences in protein domains among *CDH13* isoforms identified in the DTU analysis may directly affect muscle regenerative capacity and vascularization. Notably, one of the two enriched GO terms for DTU was adiponectin binding (GO:0055100), reinforcing this interpretation and suggesting potential molecular mechanisms through which late-gestation maternal supplementation may influence splicing regulation and exert long-term functional effects in offspring muscle.

Additional enriched terms associated with biotin (e.g., biotin binding, biotin metabolism, biotin [biotin carboxyl-carrier protein] ligase activity, and response to biotin) were affected by late gestation supplementation (PP × NP) and whole gestation supplementation (FP × NP). Biotin is a B vitamin (B7), which is an essential cofactor necessary for the activation of various carboxylase enzymes (Zempleni et al., 2008). These enzymes play key roles in fatty acids synthesis and catabolism, leucine metabolism, and gluconeogenesis (Zempleni and Kuroishi, 2012). One of the genes responsible for the differences observed in biotin metabolism is *HLCS* (*ENSBTAG00000033679*), which encodes holocarboxylase synthetase, the enzyme that catalyzes the attachment of biotin to carboxylases (León-Del-Rió et al., 2017). In our DTE analysis, *HLCS* transcript *MSTRG.802.7* was upregulated in the NP group, linked to both PP and FP contrasts, suggesting that maternal supplementation influences biotin-dependent enzyme activity in offspring muscle. The activity of these enzymes may influence metabolic efficiency as well as energy and protein metabolism in animals that receive protein-energy supplementation during gestation (PP and FP) compared with the control (NP). In our previous study investigating the long-term effects of prenatal nutrition on metabolic pathways through integrated metabolomics and metagenomics analyses (Polizel et al., 2025a), biotin-related pathways were functionally enriched in rumen fluid. These pathways exhibited distinct regulation patterns across maternal nutrition groups, as revealed by Weighted Gene Co-expression Network Analysis (WGCNA), with some modules showing opposite correlations, indicating differential regulation of biotin metabolism (Polizel et al., 2025a). This suggests that systemic shifts in biotin metabolism may occur due to differences linked to maternal nutrition.

The differences observed between PP and FP were subtle, with no functional isoform switching detected by the DTU analyses. Notably, the PP group showed upregulation of the novel transcript *MSTRG.1066.2*, within the *SLC7A8* gene, which was absent in the FP group, and may influence amino acid transport, as also indicated by ORA. Overall, however, the timing of protein-energy supplementation during gestation had only minor effects on muscle differential gene expression (DGE), DTE, DTU, and splicing dynamics.

## 5 Conclusions

Collectively, this study provides novel evidence that maternal nutrition during gestation may modulate the offspring muscle transcriptome primarily through AS and isoform regulation. Prenatal nutrition in beef cattle had a limited impact on gene expression level as revealed by differential gene expression analysis; however, more detailed transcript-level assessments (DTU and DTE) showed a greater impact of whole gestation protein–energy supplementation (FP) in comparison to the control group (NP). These transcriptomic shifts highlight the role of AS events and functional consequences mainly associated with amino acid transporters and members of the SLC superfamily. These findings indicate that transcript usage and isoform regulation provide an additional layer of molecular adaptation beyond differential gene expression, with potential long-term effects on muscle metabolism and growth efficiency. Although the overall number of significant events was modest, the consistent enrichment of processes related to amino acid and biotin metabolism suggests that maternal nutrition strategies can shape long-term offspring physiology through subtle but functionally meaningful transcript-level regulation. By applying more complex bioinformatics analyses, such as DTU and DTE, our study advances beyond traditional gene-level approaches and helps to address important gaps in the literature, offering new perspectives on how maternal nutrition can shape long-term molecular regulation in offspring muscle.

## Data Availability

The datasets presented in this study can be found in online repositories. The transcriptome dataset analyzed during the current study is available in the European Nucleotide Archive (ENA) repository (EMBL-EBI), under accession number PRJEB84398 (https://www.ebi.ac.uk/ena/browser/view/PRJEB84398).

## Supplementary material

Additional File S1 Annotation file with known transcripts identified.

Additional File S2 Annotation file with novel genes and transcripts identified.

Additional File S3 Annotation file with novel transcripts from reference genes identified.

Additional File S4 Annotation file with potential artifacts found.

## Glossary

A3: Alternative 3′ splice site
A5: Alternative 5′ splice site
AI: Artificial insemination
Ala: Alanine
AS: Alternative splicing
ATSS: Alternative transcription start site
ATTS: Alternative transcription termination site
BP: Biological processes
BW: Body weight
CC: Cellular component
CP: Crude protein
CPM: Counts per million
Cys: Cysteine
DGE: Differential gene expression
dIF: Difference in isoform fraction
DIO: Diet-induced obesity
DTE: Differential transcript expression
DTU: Differential transcript usage
FDR: False discovery rate
FP: Full programming
Glu: Glutamate
Gly: Glycine
GO: Gene ontology
IF: Isoform fraction
IR: Intron retention
KEGG: Kyoto Encyclopedia of Genes and Genomes
Leu: Leucine
MF: Molecular functions
NMD: Nonsense-mediated decay
NP: Not-programmed
ORA: Over-representation analysis
ORFs: Open reading frames
PCA: Principal component analysis
PP: Partial programming
REAC: Reactome
RIN: RNA integrity number
Ser: Serine
SLC: Solute Carrier superfamily
*SLC16A6*: Solute Carrier Family 16 Member 6
*SLC25A30*: Solute Carrier Family 25 Member 30
*SLC7A8*: Solute Carrier Family 7 Member 8
SoHo: Sorbin domain
*SORBS3*: Sorbin and SH3 Domain Containing 3
TDN: Total digestible nutrients
TMM: Trimmed Mean of M-values
TPM: Transcripts per million
Val: Valine
